# The role of ^99m^Tc-DPD bone SPECT/CT in the management of growth disturbance of the long bones in pediatric patients: a retrospective observational study

**DOI:** 10.1186/s12891-023-06777-0

**Published:** 2023-08-24

**Authors:** Chang Ho Shin, Wonseok Whi, Yoon Joo Cho, Won Joon Yoo, In Ho Choi, Gi Jeong Cheon, Tae-Joon Cho

**Affiliations:** 1 Division of Paediatric Orthopaedics, Seoul National University Children’s Hospital, 101 Daehak-ro, Jongno-gu, Seoul, 03080 Republic of Korea; 2 Department of Orthopaedic Surgery, Seoul National University College of Medicine, 101 Daehak-ro, Jongno-gu, Seoul, 03080 Republic of Korea; 3 Department of Molecular Medicine and Biopharmaceutical Sciences, Seoul National University, 1 Gwanak-ro, Gwanak-gu, Seoul, 08826 Republic of Korea; 4 Department of Nuclear Medicine, Seoul National University Hospital, Seoul National University College of Medicine, 101 Daehak-ro, Jongno-gu, Seoul, 03080 Republic of Korea

**Keywords:** Bone SPECT/CT, Growth disturbance, Deformity, Growth plate, Physis

## Abstract

**Backgrounds:**

Determining the precise localization of diseased physes is crucial for guiding the treatment of growth disturbances. Conventional radiography, computed tomography (CT), and magnetic resonance imaging only provide information on physeal anatomy. Planar bone scintigraphy and bone single-photon emission computed tomography (SPECT) resolutions are suboptimal for clinically managing growth disturbances. Bone SPECT/CT, which provides high-resolution functional information, can be a useful tool for evaluating growth disturbances. The purposes of this study were to identify the conditions in which bone SPECT/CT outperforms planar scintigraphy or SPECT for evaluating the location and activity of diseased physes and to assess surgical outcomes using bone SPECT/CT findings in pediatric patients experiencing long bone growth disturbances.

**Methods:**

Fifty-nine patients who underwent bone SPECT/CT between January 2018 and January 2021 to evaluate physeal activity using technetium-99 m-labeled 2,3-dicarboxypropane-1,1-diphosphonate (^99m^Tc-DPD) were included. The proportions of patients for whom certain modalities provided sufficient data for selecting treatment plans for growth disturbances were compared based on the site of the diseased physis, growth disturbance cause, and shape of deformity (i.e., SPECT/CT vs. planar scintigraphy and SPECT/CT vs. SPECT). For assessing surgical outcomes, progression of post-surgical deformity was investigated by measuring the angles reflecting the degree of deformity, iliac crest height difference, or ulnar variance on radiographs.

**Results:**

Bone SPECT/CT was sufficient for selecting a treatment plan, but planar scintigraphy or SPECT alone was insufficient in every 10 patients with diseased physes inside the femoral head (p = 0.002) and in every six with physes that were severely deformed or whose locations were unclear on conventional radiography (p = 0.03). In the proximal or distal tibia, where the tibial and fibular physes often overlapped on planar scintigraphy due to leg rotation, bone SPECT/CT was sufficient in 33/34 patients (97%), but planar scintigraphy and SPECT were sufficient in 10/34 (29%) (p < 0.001) and 24/34 (71%) patients, respectively (p = 0.004). No progression or deformity recurrence occurred.

**Conclusions:**

Bone SPECT/CT may be indicated in proximal femoral growth disturbance, when the physis is unclear on conventional radiography or severely deformed, the leg exhibits rotational deformity, or the patient is noncompliant.

**Supplementary Information:**

The online version contains supplementary material available at 10.1186/s12891-023-06777-0.

## Background

Identifying the precise localization of a diseased physis is crucial to achieve desirable outcomes when treating growth disturbances. Although conventional radiographic techniques have been used to evaluate growth disturbances, they cannot precisely determine the localization of diseased physes. Computed tomography (CT) and magnetic resonance imaging (MRI) are useful for visualizing bony bridges [1]. However, because physeal biological activity cannot be inferred from these anatomical imaging methods, they cannot identify diseased physes lacking a bone bridge.

In contrast, nuclear medicine imaging using bone-seeking agents, especially ^99m^Tc-labeled phosphate compounds, can provide physeal activity data [2–8]. However, there are limitations of planar bone scintigraphy, as it generates a projected 2D image of a 3D object and cannot provide information about the exact location, extent, and shape of a diseased physis. Bone single-photon emission computed tomography (SPECT) facilitates the examination of physeal activity with greater contrast and anatomical clarity [9]. However, the resolution of SPECT data is suboptimal for clinically managing growth disturbances, considering the small size of the physes in young children.

SPECT/CT, a hybrid imaging technique combining SPECT and CT, provides a more accurate localization of tissue function in any plane, making it useful in various clinical situations [10]. However, no studies have investigated the use of SPECT/CT for evaluating growth disturbances of long bones.

The purposes of this study were to identify the clinical conditions in which bone SPECT/CT outperforms planar scintigraphy or SPECT for evaluating the location and activity of a diseased physis and to assess the outcomes of epiphysiodesis or bone bridge resection in pediatric patients with angular limb deformities or limb length discrepancy, with treatment decisions based on bone SPECT/CT.

## Methods

This study was approved by the Institutional Review Board. The need for written informed consent was waived.

### Patients

**Fig. 1:**
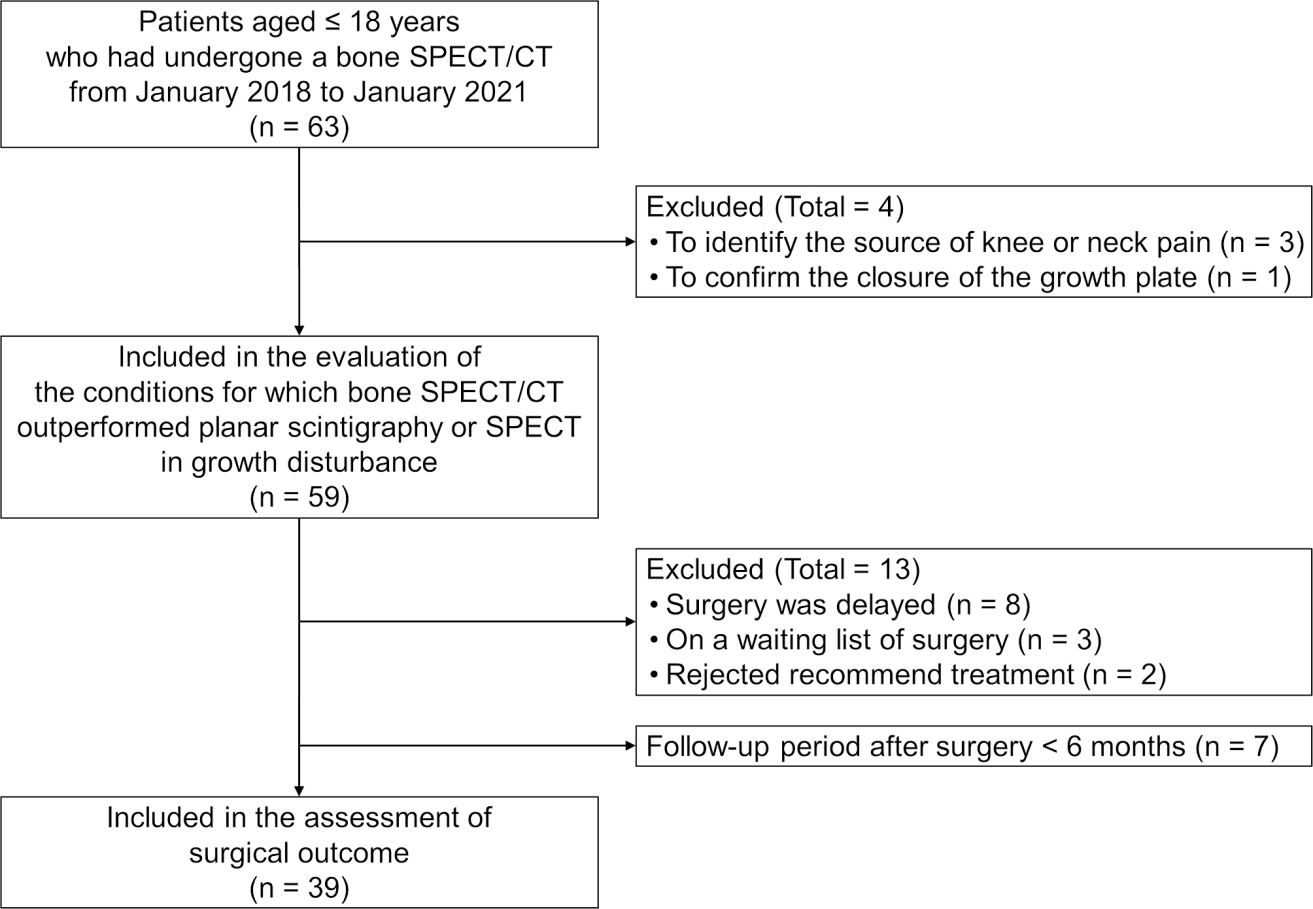
Flowchart of the study population

A clinical data warehouse of a single tertiary care pediatric center was queried to identify all patients aged 18 years and younger who underwent bone SPECT/CT between January 2018 and January 2021 (n = 63). The medical records and imaging findings were retrospectively analyzed. Three patients and one patient who underwent bone SPECT/CT to identify the source of knee or neck pain and to confirm growth plate closure, respectively, were excluded. Fifty-nine patients who underwent bone SPECT/CT to evaluate growth disturbance constituted the population (Fig. 1). All patients had undergone at least one form of conventional radiography, CT, or MRI before bone SPECT/CT. Thirty-one patients (53%, 31/59) underwent MRI combined with bone SPECT/CT to evaluate physeal status and deformity. Fifty-six patients (95%) underwent bone SPECT/CT of a single hip (n = 10), knee (n = 32), ankle (n = 10), or wrist (n = 4) joint; two patients underwent evaluations of knee and ankle joints simultaneously; and one underwent simultaneous evaluation of hip, knee, and ankle joints. No patient underwent bone SPECT/CT more than once. Fifty-four patients (92%) underwent bone SPECT/CT for suspicion of a hypoactive physis, whereas five (8%) were suspected of having a hyperactive physis (Table 1).

**Table 1:** Demographic characteristics of the patients

Characteristics	Hypoactive physis (N = 54)	Hyperactive physis (N = 5)	P value	Effect size
Age at bone SPECT/CT^*^ (years)	11.2 ± 2.5 (4.1–17.5)	11.3 ± 1.8 (9.5–13.7)	0.89^‡^	0.2∥
Female sex^†^	31 (57%)	3 (60%)	1.00^§^	0.01¶
Site of bone SPECT/CT^†^			0.02^§^	0.6¶
Hip	7 (13%)	3 (60%)		
Knee	31 (57%)	1 (20%)		
Ankle	10 (19%)	0		
Knee and ankle	2 (4%)	0		
Hip, knee, and ankle	0	1 (20%)		
Wrist	4 (7%)	0		
Cause of growth disturbance^†^			< 0.001^§^	0.8¶
Trauma	20 (37%)	0		
Skeletal dysplasia or syndromic disorder	14 (25%)	2 (40%)		
Infection	8 (15%)	0		
Tumor	8 (15%)	0		
Idiopathic	4 (7%)	0		
Developmental dysplasia of the hip	0	3 (60%)		
Presence of bone bridge^†^	27 (50%)	0	0.06^§^	0.3¶

^*^The values represent the mean and standard deviation, with ranges in parentheses. ^†^The values represent the number of patients, with percentages in parentheses. ^‡^Wilcoxon rank-sum test. ^§^Fisher’s exact test. ∥Cohen’s D. ¶Cramér’s V.

### Planar scintigraphy and bone SPECT/CT

Planar scintigraphy and SPECT/CT were performed 3 h after intravenous administration of the radiopharmaceutical ^99m^Tc-labeled 2,3-dicarboxypropane-1,1-diphosphonate (^99m^Tc-DPD), according to the European Association of Nuclear Medicine guidelines [11]. Chloral hydrate syrup was prescribed for sedation in a 4.1-year-old patient (2%). No general anesthesia was administered. No subject experienced adverse reactions to the radiopharmaceutical or sedative.

Both planar bone scintigraphy and SPECT/CT were performed by centering both joints, with a range between the midportions of the bone proximal and distal to the joint. Planar bone scintigraphy was performed using a gamma camera (Discovery NM 830, GE Healthcare, Waukesha, WI, US) with a low-energy high-resolution (LEHR) collimator. A zoom factor of 1.0 and a matrix size of 256 × 256 were utilized. Anterior and posterior views were acquired at 400 kcounts each.

SPECT/CT was performed using a hybrid dual-head gamma camera and a 16-slice CT scanner (Discovery NM/CT 670, GE Healthcare, Waukesha, WI, US). SPECT was performed using a LEHR collimator with 360° orbit, 120 steps (20 s/step), and a matrix size of 128 × 128, whereas the CT component of SPECT/CT comprised a 16-detector row helical CT scanner at 120 kVp, 40 mAs, 1 s gantry rotation, and reconstructed section thickness of 3.75 mm.

### Conditions for which bone SPECT/CT outperformed planar scintigraphy or SPECT

All radiologic images were reviewed by two authors (CHS and TJC) to make a consensus on whether the data from each nuclear imaging modality (planar scintigraphy, SPECT, or bone SPECT/CT) were sufficient for selecting a treatment plan for growth disturbance. The data was deemed sufficient for selecting a treatment plan when it provided precise information regarding the location, extent, and activity of the diseased physis, enabling an understanding of current deformities and prediction of future deformities. The proportions of patients for whom certain imaging modalities provided sufficient data were compared (i.e., bone SPECT/CT vs. planar scintigraphy and SPECT/CT vs. SPECT alone) based on the site of the diseased physis, cause of growth disturbance, and shape of deformity.

### Assessment of surgical outcomes

Surgeries were based on the location, extent, and activity of the diseased physis on bone SPECT/CT and additional information derived from conventional images. Briefly, we performed bone bridge resection only in patients with remaining physeal activity around small bone bridges. Hemiepiphysiodesis was performed in patients without bone bridges and with physeal activity on the concave side of the deformity. In patients with little or no physeal activity, procedures preventing further deformity were not performed, and surgery was only conducted to correct current deformities.

Of the 59 patients, eight experienced surgical delays because bone SPECT/CT findings predicted that the deformity would be minimal or better corrected after skeletal maturation. Three patients were on surgical waiting lists at the time of the medical record review. Two patients refused treatment. Of the remaining 46 patients, seven whose postsurgical follow-up period was < 6 months were excluded, as the time was insufficient for evaluating the deformity’s progression or recurrence. Ultimately, surgical outcomes were evaluated in the remaining 39 patients (Fig. 1) (Table 2).

**Table 2:** Demographic characteristics of the patients who underwent (hemi)epiphysiodesis or bone bridge resection

Characteristics	Permanent or temporary (hemi)epiphysiodesis (N = 31)	Bone bridge resection (N = 8)	P value	Effect size
Age at surgery^*^ (years)	11.9 ± 2.5 (4.6–17.5)	9.6 ± 2.1 (6.3–12.8)	0.01^‡^	0.9∥
Follow-up period^*^ (years)	1.4 ± 0.8 (0.5–3.6)	1.6 ± 0.5 (0.6–2.4)	0.27^‡^	0.2∥
Female sex^†^	16 (52%)	5 (63%)	0.7^§^	0.1¶
Simultaneous procedure			0.02^§^	0.5¶
Corrective osteotomy	4 (13%)	5 (63%)		
Distraction osteogenesis	1 (3%)	0		

^*^The values represent the mean and standard deviation, with ranges in parentheses. ^†^ The values represent the number of patients, with percentages in parentheses. ^‡^Wilcoxon rank-sum test. ^§^Fisher’s exact test. ∥Cohen’s D. ¶Cramér’s V.

In patients whose angular deformity was the main concern to treat, the angles reflecting the degree of deformity were compared between the preoperative and latest follow-up radiographs. The angles measured were the tibiofemoral angle (TFA) (n = 22), Hilgenreiner-epiphyseal angle (HEA) (n = 6) [12], lateral distal tibial angle (LDTA) (n = 4), and posterior distal femoral angle (PDFA) (n = 1) [13]. In patients whose limb length discrepancy was the main concern to treat, the iliac crest height difference (n = 4) or ulnar variance (n = 2) [14] was compared between the preoperative and latest follow-up radiographs. Surgical outcomes were classified as satisfactory when those angles, iliac crest height difference, or ulnar variance did not worsen and as unsatisfactory when they worsened.

To determine the intraobserver reliability of radiographic measurements, measurements were made by one of the authors (YJC) on 2 different days, 1 week apart. To determine the interobserver reliability of radiographic measurements, the same measurements were made by another author (CHS) following a consensus-building session on how to select the landmark points for measuring the parameters with the first author doing the measurements (YJC). Intra- and interobserver reliabilities were examined using intraclass correlation coefficients (ICCs) (absolute-agreement-type, single-measurement, 2-way random-effect model) (Supplemental Table 1). The interobserver reliability between the two authors (CHS and TJC) of whether bone SPECT/CT provided sufficient data for selecting treatment plans for growth disturbances was calculated using the Cohen kappa coefficient. The interobserver kappa statistic was 0.792.

### Statistical analysis

Physeal activities were classified into three types depending on ^9m^Tc-DPD uptake in the diseased physis compared with the contralateral physis: hyperactive (more uptake), normal (similar uptake), and hypoactive (less uptake). Patients with a hyperactive or hypoactive physis were compared, as were those who underwent (hemi)epiphysiodesis or bone bridge resection, using Wilcoxon rank-sum test for continuous variables and Fisher’s exact test for categorical variables. The proportions of patients for whom certain imaging modalities provided sufficient data for selecting a treatment plan were compared using McNemar’s test. Surgical outcomes were evaluated using Wilcoxon signed-rank test. Statistical significance was set at P < 0.05. Statistical analysis was performed using Stata 15.1 (StataCorp., College Station, TX, USA).

## Results

### Conditions for which bone SPECT/CT outperformed planar scintigraphy or SPECT

Overall, the proportion of patients whose bone SPECT/CT data were sufficient for selecting a treatment plan (97%, 57/59) was larger than that of planar scintigraphy (31%, 18/59) (p < 0.001) or SPECT alone (59%, 35/59) (p < 0.001).

**Fig. 2:**
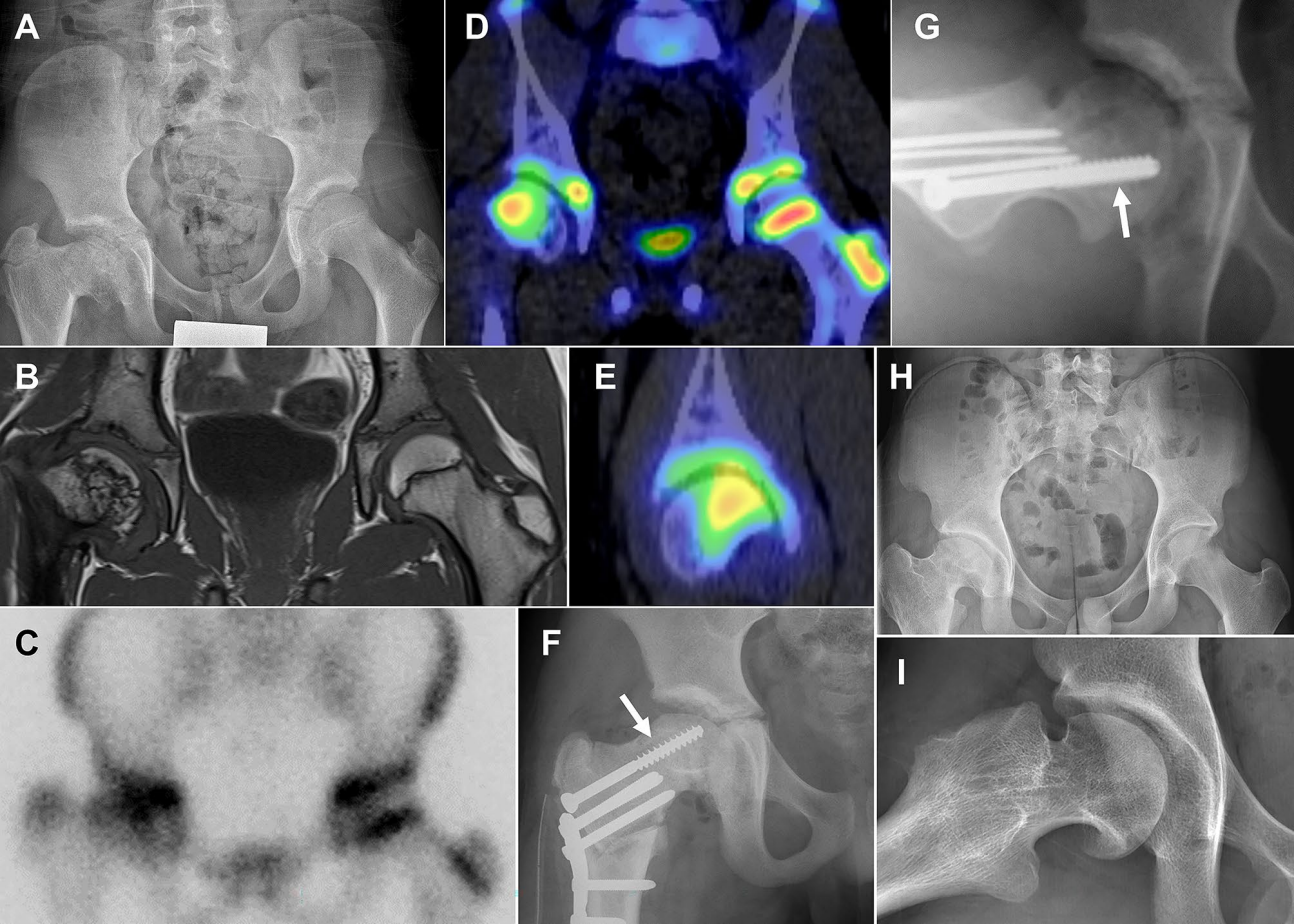
Advantage of bone SPECT/CT for evaluating growth disturbance of the proximal femur. A standing anteroposterior hip radiograph of a 10-year-old boy shows right coxa vara recurrence following proximal femoral valgus osteotomy due to septic hip sequelae (**A**). On T1-weigthed MRI, the physis exhibits irregular-contour, but a bone bridge is not definitely identified. (**B**). The proximal femoral physis is inseparable from the acetabular cartilage on planar scintigraphy derived from SPECT/CT (**C**) but is distinguishable on fused SPECT/CT (**D** and **E**). On bone SPECT/CT, the uptake of ^99m^Tc-DPD is evident only at the posterolateral (**D** and **E**) aspects in the right proximal femoral physis, which is considered the cause of recurrent deformity. A step-cut proximal femoral valgus osteotomy was performed along with the insertion of a transphyseal screw (arrows) in the posterolateral physis to prevent recurrence of coxa vara (**F** and **G**). No recurrence was noted until skeletal maturity (**H** and **I**)

At the growth plate inside the femoral head, bone SPECT/CT was sufficient for selecting a treatment plan in all 10 patients (100%, 10/10), but planar scintigraphy or SPECT was insufficient in every patient (0%, 0/10) (Fig. 2). The proportion of patients for whom SPECT/CT was sufficient for selecting a treatment plan in the femoral head was significantly larger than that of planar scintigraphy (p = 0.002) or SPECT (p = 0.002).

**Fig. 3:**
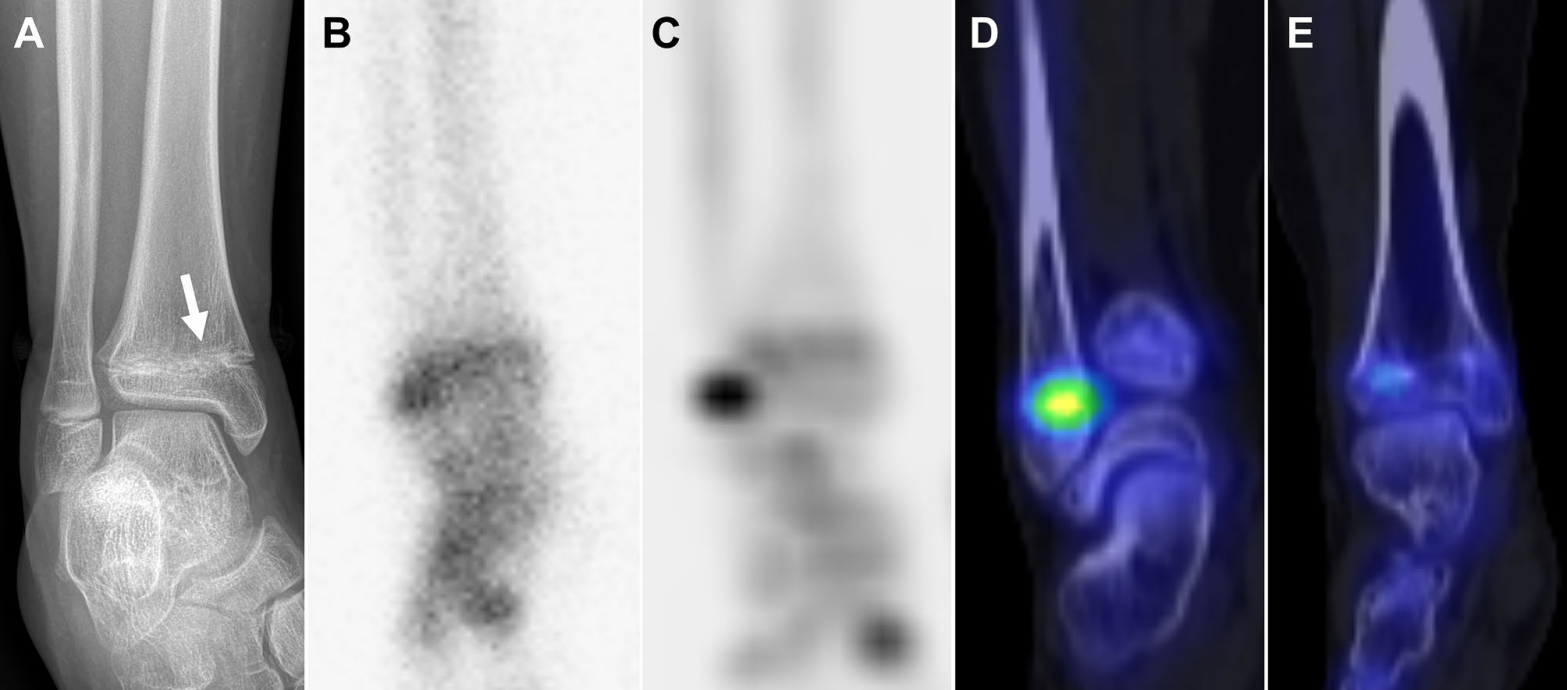
Advantage of bone SPECT/CT for assessing overlapping lateral tibial and fibular physes. A bone bridge (arrow) is suspected in the medial distal tibial physis on ankle mortise radiograph in an 11.4-year-old boy who sustained a physeal fracture (**A**). Bone SPECT/CT was performed to assess the growth potential in the remaining physis surrounding the bone bridge. On planar scintigraphy derived from SPECT/CT, the physeal activity of the distal tibia is unclear, due to the overlap of the distal fibular and lateral distal tibial physes (**B**). Differentiation of these two physes is possible on SPECT (**C**), although it is clearer on fused SPECT/CT (**D** and **E**). SPECT and SPECT/CT indicate physeal activity only in the distal fibular physis, whereas activity is absent in the near entire distal tibial physis. Based on these findings on SPECT/CT, epiphysiodesis was performed in the right distal fibula rather than bone bridge resection in the right distal tibia

**Fig. 4:**
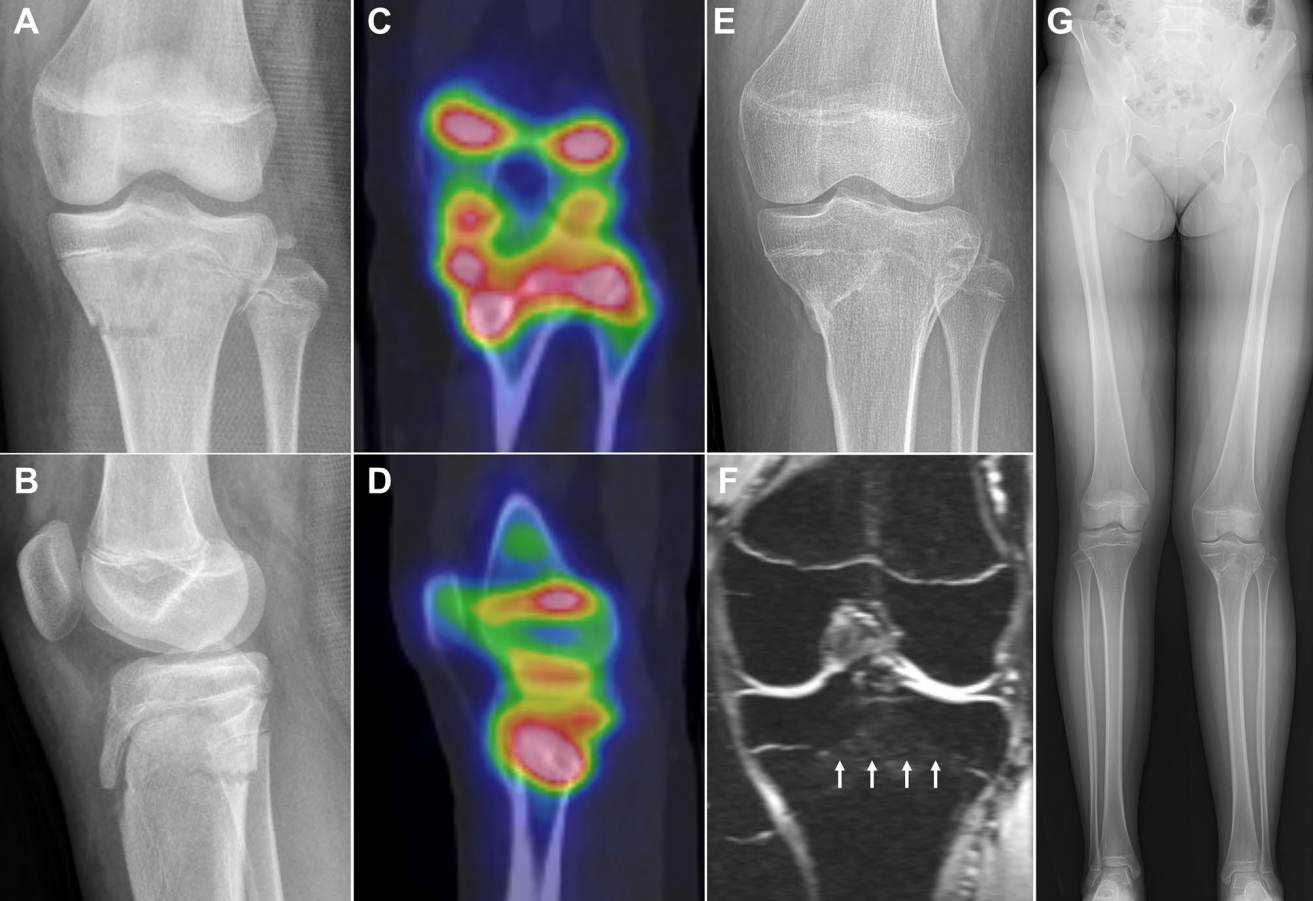
A case of a girl demonstrating insufficiency of bone SPECT/CT in selecting a treatment plan. She had a left proximal tibial physeal fracture at the age of 10.8 years (**A** and **B**). Bone SPECT/CT performed at post-operative 6 months reveals hyperactivity of the overall proximal tibial physis, contrary to our expectation of hypoactivity in some areas of the physis (**C** and **D**). Growth disturbance is suspected on knee radiograph at post-operative 10 month (**E**). She underwent permanent epiphysiodesis of the proximal tibia and fibula due to extensive bone bridge (arrows) (**F**) and progressive limb length discrepancy (**G**). She plans to undergo tibial and fibular lengthening after reaching skeletal maturity

In the proximal or distal tibia, tibial and fibular physes often overlapped on planar scintigraphy due to leg rotation caused by deformity or patient noncompliance. Even on planar scintigraphy performed with the patella facing forward, the lateral aspect of the tibial physis frequently overlapped with the fibular physis; differentiating between the two was often possible on SPECT, although it was clearer on SPECT/CT (Fig. 3). Therefore, bone SPECT/CT was sufficient for selecting a treatment plan in 33/34 patients (97%), whereas planar scintigraphy and SPECT were sufficient in only 10/34 (29%) and 24/34 (71%) patients, respectively. The proportion of patients for whom SPECT/CT was sufficient for selecting a treatment plan in the proximal or distal tibia was significantly larger than that of planar scintigraphy (p < 0.001) or SPECT (p = 0.004). Bone SPECT/CT was insufficient in a patient suspected of having a hypoactive physis due to development of a bone bridge after proximal tibial physeal fracture because bone SPECT/CT revealed hyperactivity of the overall proximal tibial physis (Fig. 4).

**Fig. 5:**
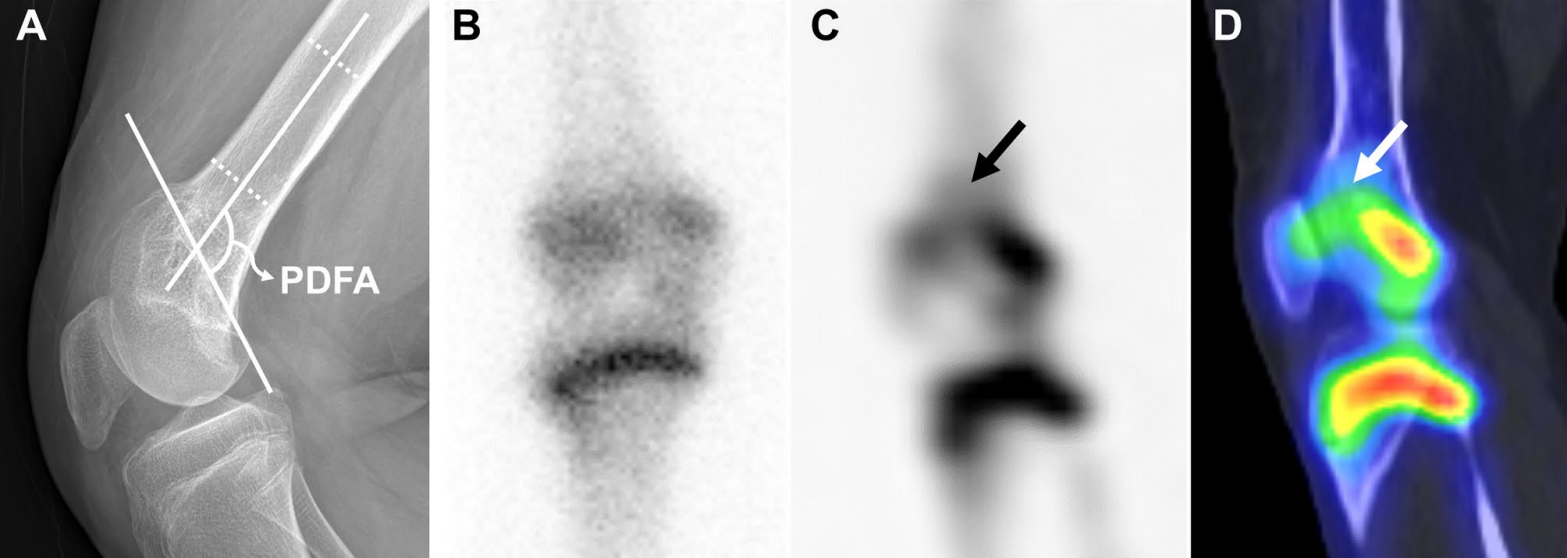
Advantage of bone SPECT/CT for assessing physeal status in which sagittal plane deformity is apparent. A 10.7-year-old girl presented with genu recurvatum after right distal femoral physeal fracture. Extension deformity of the distal femur is identified with a an anatomic posterior distal femoral angle of 125°, although the precise physeal location is obscure on conventional radiography (**A**). On anterior view planar scintigraphy, an overall decrease in distal femoral physeal activity is apparent. (**B**). However, hypoactivity is limited to the anterior distal femoral physis on the sagittal SPECT image, which has caused extension deformity (arrows) (**C**). It is more clearly visualized on the sagittal fused SPECT/CT (**D**). PDFA = posterior distal femoral angle

In the distal femur, with a relatively long and horizontal physis, the proportion of patients for whom SPECT/CT was sufficient for selecting a treatment plan (100%, 11/11) was not significantly different from that of planar scintigraphy (73%, 8/11) (p = 0.25) or SPECT (100%, 11/11) (p = 1.00). All three patients for whom bone SPECT/CT and SPECT were sufficient but planar scintigraphy was not, had diseased distal femoral physes located anteriorly or posteriorly, causing sagittal plane deformity (Fig. 5). Anterior and posterior planar scintigraphy images were sufficient for assessing the status of physes causing coronal plane deformity or limb length discrepancy.

**Fig. 6:**
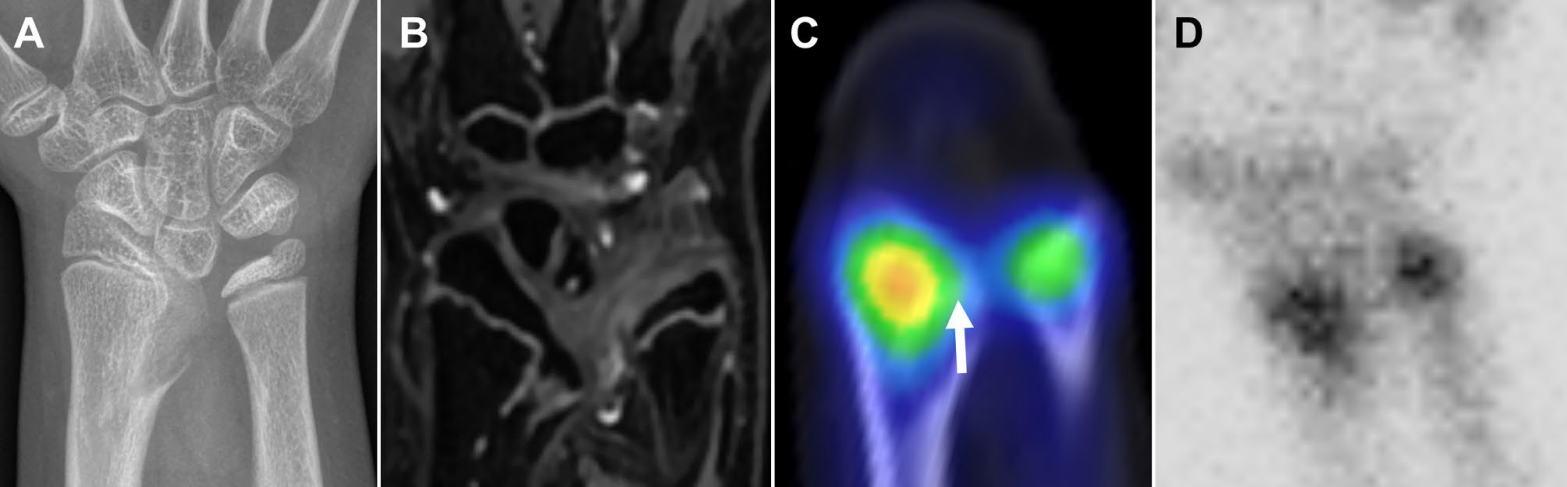
Advantage of bone SPECT/CT for evaluating growth disturbance of the small longitudinal bone. A 10.6-year-old girl with Leri-Weill dyschondrosteosis has Madelung’s deformity in both wrists (**A**). A physeal bar is not definitely identified on gradient-recalled-echo T2-weighted MRI (**B**). The ulnar side of the distal radial physis (arrow) is hypoactive compared with the radial side, suggesting that the deformity will aggravate over time (**C**). This discrepancy in physeal activity between the ulnar and radial sides is not evident on planar scintigraphy (**D**)

In the distal radius, where the physes were relatively small, bone SPECT/CT was sufficient in 3/4 patients (75%), but planar scintigraphy or SPECT was sufficient in none (0/4, 0%) (Fig. 6). The proportion of patients for whom SPECT/CT was sufficient for selecting a treatment plan in the distal radius was not significantly different from that of planar scintigraphy (p = 0.25) or SPECT (p = 0.25). Discrimination between ^99m^Tc-DPD uptake into physes or carpal bones was difficult, even on bone SPECT/CT in one patient.

**Fig. 7:**
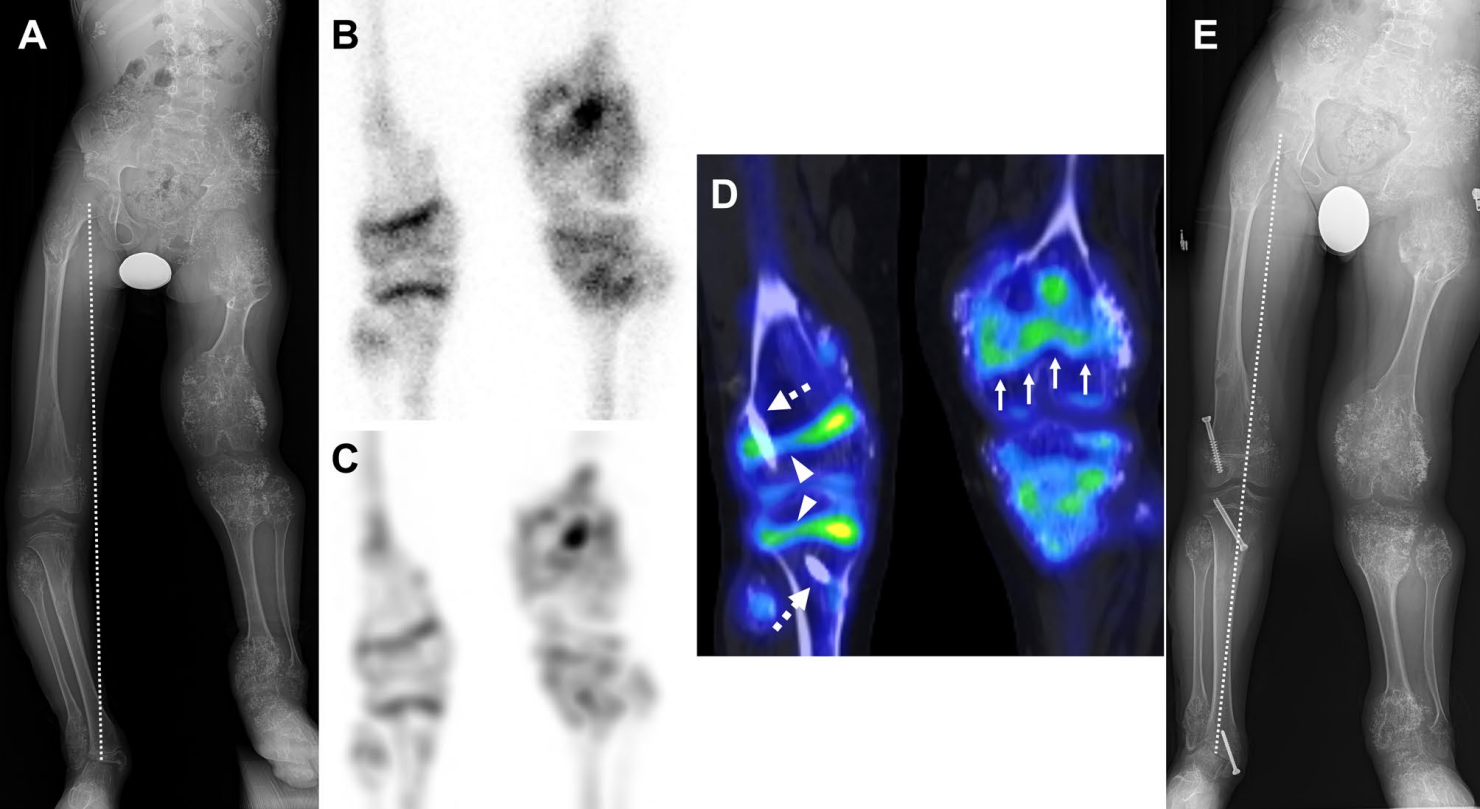
Advantage of bone SPECT/CT for evaluating physes whose locations are unclear on conventional radiographs. A 9.6-year-old boy with metaphyseal chondromatosis with D-2-hydroxyglutaric aciduria exhibited shortening and genu valgum on the left side and genu varum on the right side (**A**). The locations of the physes of the left leg are vague on conventional radiography (**A**). It is difficult to evaluate physeal activity of the left leg on planar scintigraphy (**B**) and SPECT (**C**) because uptake of ^99m^Tc-DPD can occur both in the tumor and physis, and the physis may be deformed. The uptake of the radiotracer by the tumor and physis can be differentiated on fused SPECT/CT, owing to its high resolution and anatomic information (**D**). Physeal activity in the left distal femur (arrows) is identified despite a decrease (**D**). Screws inserted for temporary hemiepiphysiodesis (dashed arrows) have resulted in hypoactive lateral physes (arrowheads) compared to the normal activity of the medial physis of the right knee (**D**). The physeal activity of the right knee indicates that genu varum of the right leg will improve (**A** and **E**)

Bone SPECT/CT was sufficient in every six patients (100%, 6/6) who had a physis whose location was unclear on conventional radiography or severely deformed due to skeletal dysplasia, and in those who had a tumor or had undergone previous chemotherapy (Fig. 7), whereas neither planar scintigraphy nor SPECT (0%, 0/6) was sufficient. The proportion of patients for whom SPECT/CT was sufficient for selecting a treatment plan when the physis was unclear on conventional radiography or severely deformed was significantly larger than that of planar scintigraphy (p = 0.03) or SPECT (p = 0.03).

**Fig. 8:**
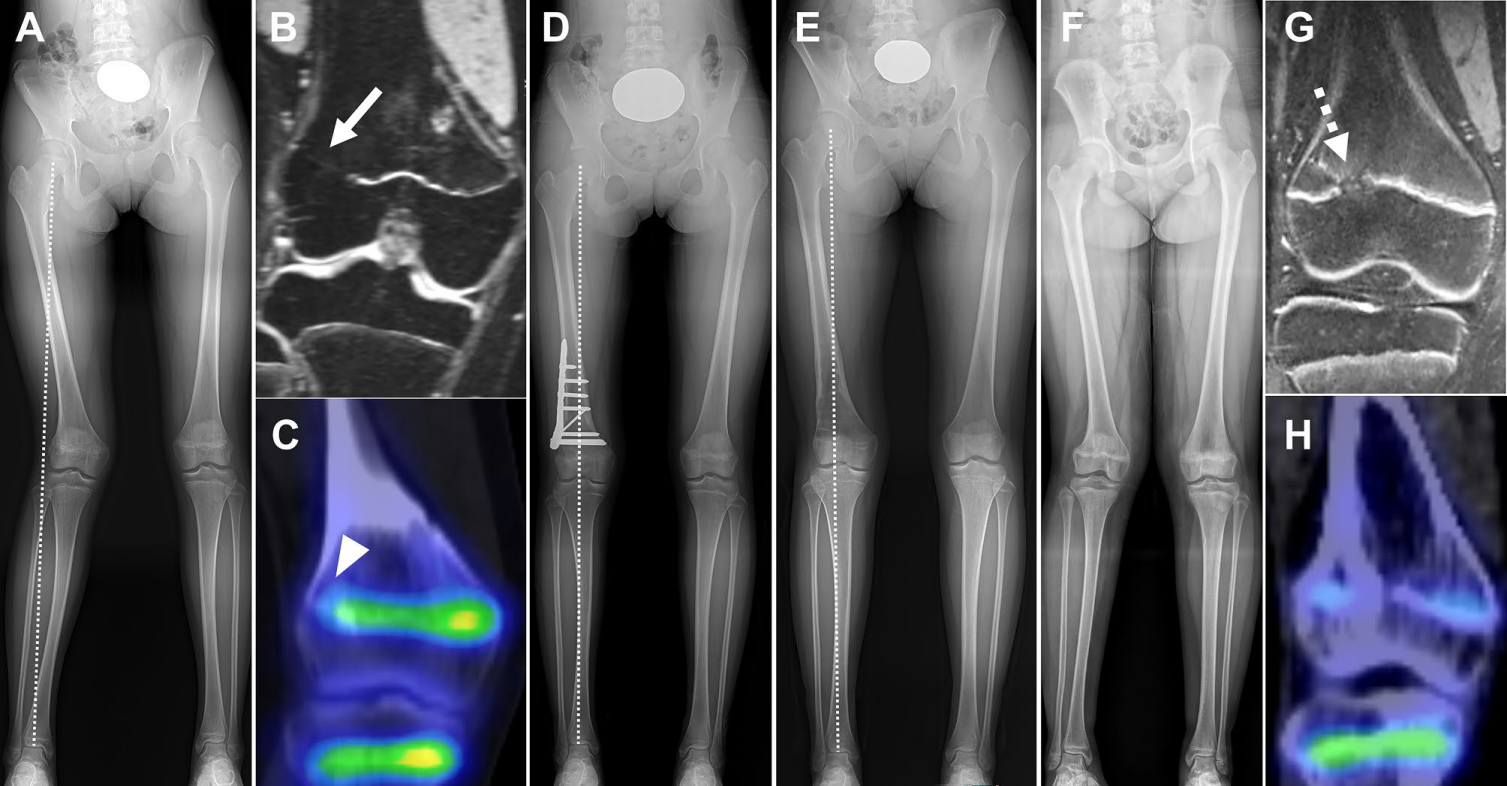
Bone SPECT/CT-based treatment planning for growth disturbance of the long bone. An 11-year-old girl presented with posttraumatic genu valgum and shortening of the right leg (**A**). MRI reveals a peripheral bone bridge (arrow) at the lateral distal femoral physis (**B**). There is no uptake of ^99m^Tc-DPD at the bone bridge site (arrowhead) on bone SPECT/CT (**C**); however, physeal activity remains in the surrounding physis (**C**). She underwent arthroscopy-assisted bone bridge resection and opening-wedge distal femoral varus osteotomy (**D**). No aggravating leg length discrepancy was noted until post-operative 2.3 years, indicating that the right leg had undergone longitudinal growth (**E**). Another 11-year-old girl presented with posttraumatic genu valgum and shortening of the right leg (**F**). MRI reveals a slightly laterally located bone bridge (dashed arrow) at the distal femoral physis (**G**). On bone SPECT/CT, almost no remaining physeal activity is apparent in the entire right distal femoral physis (**H**). Based on these findings, she underwent distraction osteogenesis rather than epiphysiodesis or bone bridge resection

Even in patients with a bone bridge observed on MRI, SPECT/CT aids in surgical planning by evaluating the activity of an apparently intact physis surrounding the bone bridge (Fig. 8).

### Assessment of surgical outcomes

All 39 patients exhibited satisfactory surgical outcomes, with no progression or recurrence of deformity visible on radiographs (Table 3).

**Table 3:** Surgical outcomes of the patients (N = 39)

Parameters*	Preoperative	Latest follow-up	P value^†^	Amount of correction	Effect size
TFA^‡^ (degrees) (N = 22)	0.5 ± 12.0 (-16–20)	0 ± 6.2 (-15–10)	0.82	8.6 ± 8.1 (0–27)	0.04∥
HEA (degrees) (N = 6)	30.5 ± 35.9 (0–78)	29.2 ± 24.1 (4–64)	0.92	8.5 ± 9.7 (0–25)	0.04∥
LDTA (degrees) (N = 4)	104.8 ± 7.0 (98–114)	96 ± 2.7 (94–100)	0.07	8.8 ± 8 (1–19)	1.6∥
PDFA (degrees) (N = 1)	119	115	N/A	4	N/A
LLD (mm) (N = 6)	-11.5 ± 12.8 (-37–-3)	-7.3 ± 8.4 (-23–0)	0.09	4.2 ± 5.9 (0–14)	0.4∥

^*^The values represent the mean and standard deviation, with ranges in parentheses. ^†^Wilcoxon signed-rank test. ^‡^A valgus TFA was given a positive value, whereas a varus TFA was given a negative value. TFA = tibiofemoral angle, HEA = Hilgenreiner-epiphyseal angle, LDTA = lateral distal tibial angle, PDFA = posterior distal femoral angle, LLD = limb length discrepancy, and N/A = not applicable. ∥Cohen’s D.

## Discussion

Deformity caused by growth disturbance in pediatric patients differs from that in adults in that it usually progresses as growth occurs. To prevent this progression, knowing the exact localization and evaluating the activity of diseased physes is of importance. Although various imaging modalities have guided the appropriate management of growth disturbances, each has certain limitations. To date, no studies have evaluated the use of bone SPECT/CT in managing growth disturbances of the long bones. This study clearly demonstrated that bone SPECT/CT is a useful diagnostic tool for localizing and evaluating the activity of diseased physes.

Traditionally, bone bridges were localized using radiographs, CT, and/or MRI [15–19]. On radiographs, bone bridges appear as focal areas of increased radiodensity bridging the physis, and/or Harris growth arrest lines are seen to converge at a bone bridge [17, 18]. On coronal and sagittal CT images that offer better contrast resolution than that of radiographs, bone bridges are visualized as a high-attenuation lesion within a low-attenuation growth plate [17]. Borsa et al. revealed that 3D rendered and projection physeal maps from MRI data are advantageous for preoperative planning [15]. We localized bone bridges in the physis based on information from traditional imaging and bone SPECT/CT.

Bone SPECT/CT has advantages over MRI in evaluating physeal status. First, it enables the evaluation of the actual physeal activity [20]. Cartilage-sensitive MRI sequences, such as gradient-recalled-echo images, facilitate a high degree of contrast between the bright cartilage and dark bone tissue, and are useful for identifying areas of bone bridge formation [1]. Whereas growth disturbances caused by posttraumatic hyperactive physis cannot be detected on MRI in the absence of a distinct bone bridge, bone SPECT/CT can provide information on the physeal activity and predict future deformity. Second, bone SPECT/CT is especially useful in patients with skeletal dysplasia, as it is often uncertain whether a physis has residual growth potential, regardless of its appearance on MRI. Third, bone SPECT/CT is also advantageous for evaluating physeal status in patients with metal implants because SPECT/CT is less affected by metal artifacts than MRI (Fig. 7).

In this study, several conditions were identified in which bone SPECT/CT could be used to evaluate the location and activity of diseased physes. First, in the proximal femur, the spherical shape of the femoral head obstructs the location of diseased physes on conventional radiography, planar scintigraphy, and SPECT; therefore, bone SPECT/CT excels in evaluating physeal status in the proximal femur. Second, bone SPECT/CT can be indicated in patients with physes whose locations are unclear on conventional radiography or in those with severely deformed bone tissue from various causes such as skeletal dysplasia or tumors. In these patients, it is difficult to combine planar scintigraphy or SPECT images with conventional images to mentally reconstruct 3-dimensional anatomy. Third, bone SPECT/CT could be indicated in patients with rotational deformity of the leg or in noncompliant patients for whom it is necessary to distinguish between fibular and lateral tibial physes. Multiplanar reconstruction of bone SPECT/CT images can overcome the problem of overlap of the fibula and tibia on planar scintigraphy. However, in patients with simple coronal plane deformity or limb length discrepancy, planar scintigraphy is sufficient for evaluating a diseased physis, especially in large bones such as the distal femur. Sagittal plane deformities in the distal femur can also be sufficiently evaluated using SPECT.

The surgical outcomes based on the bone SPECT/CT findings were satisfactory in all patients. Several studies have reported satisfactory outcomes following bone bridge resection [21, 22], whereas others reported that bone bridge re-resection was required in 13/48 patients (27%) [23], and one in five patients experienced no improvement after bone bridge resection [24]. Hasler and Foster reported that 14 of 22 patients (64%) exhibited only fair or poor outcomes following bone bridge resection [16]. In another study investigating bone bridge resection based on physeal bar mapping by CT data, 11 of 19 patients (58%) demonstrated significant improvement in mechanical axis deviation [19]. In the present study, with an average follow-up time of 1.6 years, no repeated bone bridge resection was needed, possibly due to reduced indication for bone bridge resection following thorough evaluation using bone SPECT/CT.

Bone SPECT/CT has certain disadvantages. First, it results in more radiation exposure than either SPECT or CT. Considering that growth disturbances affect children who are more susceptible to radiation than adults, the amount of clinical information that could be obtained should be weighed against any harm associated with such exposure. Bone SPECT/CT is not routinely performed for patients with growth disturbance. Fortunately, owing to technological advances, the resolution of low-dose CT has improved over time, although there is an ongoing effort to develop a more sensitive detector that requires administration of smaller amounts of radiolabeled tracers. Second, because of individual differences in physeal biological activity, radiotracer uptake must be compared between both sides. Therefore, the field of view of bone SPECT/CT must be adequate to comprise both extremities, resulting in a lower resolution for the evaluation of each physis. MRI is useful for providing complementary information in patients for whom precise identification of the physeal shape is important.

The reason why bone SPECT/CT revealed hyperactivity of the overall proximal tibial physis in the patient with bone bridges remains unclear (Fig. 4). In this specific case, the patient underwent bone SPECT/CT 6 months after the proximal tibial fracture. We presumed that the active ossification process for fracture union and bone bridge formation caused hyperactivity in the physis rather than hypoactivity due to high accumulation of bone-seeking Tc-99 m phosphorous compounds in the bone mineralization surfaces and areas with high blood flow [25]. Therefore, we do not recommend bone SPECT/CT for visualizing the diseased physes in the acute healing stage of fractures.

This study had several limitations. First, because the sample consisted of patients with heterogeneous diseases treated with various surgical interventions, it was difficult to define uniform diagnostic and outcome measures to be applied in assessing the superiority of one imaging method over another. Despite our efforts to conduct a consensus-building session, identification of the conditions in which bone SPECT/CT outperformed planar scintigraphy or SPECT lacked quantitative parameter related to the data sufficiency, making it somewhat subjective. Second, in cases of no uptake of the radiolabeled tracer, it was impossible to confirm whether a physis with a bone bridge had lost its growth activity permanently or temporarily, as it might become reactivated after untethering and decompression during bone bridge resection. Longitudinal follow-up radiography or bone SPECT/CT after bone bridge resection could provide further insights. Third, the mean age at the time of bone SPECT/CT was 11.2 years. Because the sizes of physes are smaller in younger patients, the finding of satisfactory surgical outcomes based on bone SPECT/CT may not be generalizable to very young patients.

## Conclusions

Bone SPECT/CT can help accurately visualize the location and activity of diseased physes on multiple planes. Bone SPECT/CT may be indicated when there is growth disturbance of the proximal femur, the physis is unclear on conventional radiography or severely deformed, when there is rotational deformity of the leg, or in noncompliant patients. Reducing radiation from radiopharmaceuticals and CT would expand the indications of bone SPECT/CT.

### Electronic supplementary material

Below is the link to the electronic supplementary material.

Supplementary Material 1

Supplementary Material 2

## Data Availability

Data are available from the corresponding author upon reasonable request and after ethical permissions.

## Abbreviations

^99m^Tc-DPD: Technetium-99 m-labeled 2,3-dicarboxypropane-1,1-diphosphonate
CT: Computed tomography
HEA: Hilgenreiner-epiphyseal angle
ICC: Intraclass correlation coefficient
LDTA: Lateral distal tibial angle
LEHR: Low-energy high-resolution
MRI: Magnetic resonance imaging
PDFA: Posterior distal femoral angle
SPECT: Single-photon emission computed tomography
TFA: Tibiofemoral angle
