# Supplementary material for: The role of 99mTc-DPD bone SPECT/CT in the management of growth disturbance of the long bones in pediatric patients: a retrospective observational study
Source: BMC Musculoskelet Disord. 2023 Aug 24;24:668. doi: 10.1186/s12891-023-06777-0 (PMC10464403; doi:10.1186/s12891-023-06777-0)
Supplement: Supplementary file 1 — Supplementary Material 1 [file 12891_2023_6777_MOESM1_ESM.docx]

| **Supplemental Table 1.** Intraclass correlation coefficients calculated to evaluate intraobserver and interobserver reliability | | |
| --- | --- | --- |
| **Measurements** | **Intraobserver (95% CI)** | **Interobserver (95% CI)** |
| Angles on the preoperative radiographs^*^ | 0.996 (0.989–0.998) | 0.994 (0.983–0.998) |
| Angles on the latest follow-up radiographs^*^ | 0.993 (0.987–0.997) | 0.992 (0.983–0.996) |
| LLD on the preoperative radiographs^†^ | 0.997 (0.980–1.000) | 0.998 (0.988–1.000) |
| LLD on the latest follow-up radiographs^†^ | 0.968 (0.820–0.995) | 0.955 (0.714–0.994) |
| ^*^The tibiofemoral angle (n=22), Hilgenreiner-epiphyseal angle (n=6), lateral distal tibial angle (n=4), or posterior distal femoral angle (n=1) was measured. †The iliac crest height difference (n=4) or ulnar variance (n=2) was measured. LLD = limb length discrepancy and CI = confidence interval. | | |
